# Altered Ocular Surface Temperature in Congenital Aniridia with *PAX6* Pathogenic Variants: Impact of Age, Salzmann Nodules and Ocular Surgery

**DOI:** 10.3390/life16020238

**Published:** 2026-02-02

**Authors:** Orsolya Németh, Annamária Náray, Mária Csidey, Klaudia Kéki-Kovács, Krisztina Knézy, Mária Bausz, Andrea Szigeti, Anita Csorba, Kitti Kormányos, Ditta Zobor, Zoltán Zsolt Nagy, Marta Cortón, Eszter Jávorszky, Kálmán Tory, Erika Maka, Timo Eppig, Achim Langenbucher, Nóra Szentmáry

**Affiliations:** 1 Department of Ophthalmology, Semmelweis University, 1085 Budapest, Hungary; annamaria.naray@gmail.com (A.N.); mcsidey@yahoo.com (M.C.); kovacs.klaudia94@gmail.com (K.K.-K.); knezykriszta@yahoo.com (K.K.); bauszmaria@t-online.hu (M.B.); szigandi@gmail.com (A.S.); csorbani@gmail.com (A.C.); kitti0820@gmail.com (K.K.); zobor.ditta@semmelweis.hu (D.Z.); zoltan.nagy100@gmail.com (Z.Z.N.); dr.maka.erika@gmail.com (E.M.); nszentmary@gmail.com (N.S.); 2 Department of Ophthalmology, Markusovszky University Teaching Hospital, 9700 Szombathely, Hungary; 3 Dr. Rolf M. Schwiete Center for Limbal Stem Cell and Congenital Aniridia Research, Saarland University, 66424 Homburg, Germany; 4 Heim Pál National Pediatric Institute, 1089 Budapest, Hungary; 5 Department of Genetics and Genomics, Instituto de Investigación Sanitaria-Fundación Jiménez Díaz University Hospital, Universidad Autónoma de Madrid (IIS-FJD, UAM), 28015 Madrid, Spain; mcorton@quirosalud.es; 6 Center for Biomedical Network Research on Rare Diseases (CIBERER), Instituto de Salud Carlos III, 28029 Madrid, Spain; 7 MTA-SE Lendület Nephrogenetic Laboratory, Hungarian Academy of Sciences, 1083 Budapest, Hungary; javorszky.eszter@semmelweis.hu (E.J.); tory.kalman@semmelweis.hu (K.T.); 8 1st Department of Pediatrics, Semmelweis University, 1083 Budapest, Hungary; 9 Experimental Ophthalmology, Saarland University, 66424 Homburg, Germany; timo.eppig@uni-saarland.de (T.E.); achim.langenbucher@uks.eu (A.L.)

**Keywords:** congenital aniridia, ocular thermography, Tomey TG-1000, aniridia associated keratopathy

## Abstract

*PAX6* haploinsufficiency-related congenital aniridia is frequently associated with ocular surface disease, including meibomian gland dysfunction (MGD), dry eye, limbal stem cell deficiency (LSCD), aniridia-associated keratopathy (AAK), and inflammation. This study measured ocular surface temperature (OST) at the corneal center and four paracentral points (2 mm from center) in patients with congenital aniridia and examined factors influencing OST. Forty-five eyes from 26 aniridia patients (55.6% female; 26.29 ± 17.78 years) with *PAX6* pathogenic variants and 47 eyes from 25 controls (68.1% female; 24.81 ± 4.73 years; *p* = 0.1639) were included. Body temperature, OSDI, and OST (TG-1000) were recorded; clinical assessment evaluated MGD, LSCD, AAK, iris malformation, epithelial defects, Salzmann nodules, glaucoma and previous ocular surgery. Body temperature and OSDI did not differ in aniridia and controls (*p* ≥ 0.606). LSCD was mainly Grade 2 (46.7%) or Grade 4 (40.0%), and AAK Grade 1 (33.3%) or Grade 2 (31.1%). MGD affected 51.1%, Salzmann nodules 22.2%, epithelial defects 2.2%, glaucoma 60.0%, and previous ocular surgery 35.5%. Superior OST was higher in aniridia (34.98 ± 0.55 °C vs. 34.75 ± 0.47 °C; *p* = 0.012). Exploratory univariate analyses identified that higher AAK grade correlated with lower inferior OST (*p* = 0.030), iris malformation with reduced central/paracentral OST (*p* ≤ 0.029), and Salzmann nodules with lower OST overall (*p* ≤ 0.011). However, in a multivariate model, age, Salzmann nodular degeneration, and prior ocular surgery emerged as key determinants of OST. OST may serve as a noninvasive biomarker in congenital aniridia.

## 1. Introduction

Ocular Surface Temperature (OST) has been studied for over 50 years, with early research establishing that OST is influenced by both external and internal factors. External factors include ambient temperature [1], humidity [2], wind [3], and blinking [4]. Internal factors, such as tear film dynamics and aqueous humour circulation, also play significant roles [5,6]. The quality and quantity of the tear film affect OST primarily through evaporative cooling, while the aqueous humour contributes to heat transfer via conduction and convection. In addition, aqueous humour temperature is determined by ciliary body blood flow and retrobulbar haemodynamics [7]. Furthermore, limbal vasculature may influence the OST in the limbal region [8]. The impact of corneal thickness on OST remains controversial, with conflicting results reported in various studies [9,10,11].

OST has been investigated in several ocular pathologies. In patients with dry eye disease, OST is typically elevated and decreases more rapidly during sustained eye opening compared to healthy individuals [12]. In allergic conjunctivitis, Hara et al. (2014) reported an increase in basal OST, along with a positive correlation between temperature elevation and conjunctival hyperemia [13]. In cases of corneal ulceration, OST is higher, and the degree of infection appears to correlate directly with the temperature increase [14]. A similar relationship was found in acute corneal transplant rejection, where Sniegowski et al. observed elevated OST associated with graft rejection [15]. In patients with pterygium and dry eye, Gonnermann et al. found no significant difference in average OST compared to healthy controls; however, the temperature profile during 10 s of sustained eye opening differed significantly in both the pterygium and dry eye groups when compared to the healthy group [16]. Vannetti et al. (2014) demonstrated that OST significantly increases following induced peripheral vasoconstriction, suggesting a link between systemic vascular regulation and ocular surface temperature [17]. OST measurements in glaucoma patients have yielded conflicting results, with some studies reporting higher [18] and others reporting lower OST [19]. Notably, Gugleta (1999) and Galassi et al. (2007) found correlations between OST and Doppler parameters of the ophthalmic and posterior ciliary arteries, suggesting that OST may serve as a potential marker of impaired blood supply to the optic nerve head in glaucoma [7,20].

Congenital aniridia is a rare, panocular, autosomal dominant inherited disease primarily characterized by iris hypoplasia. Nevertheless, due to variants in the *PAX6* gene, the development of nearly all ocular structures is affected. Hallmark features include progressive limbal stem cell deficiency (LSCD) and a chronic ocular surface and corneal disease known as aniridia-associated keratopathy (AAK). Other commonly associated conditions are glaucoma, cataract, lens subluxation, strabismus, as well as optic nerve coloboma and optic nerve hypoplasia, foveal hypoplasia and nystagmus [21].

In patients with congenital aniridia, multiple factors may contribute to changes in OST. Meibomian gland dysfunction has been reported in aniridia, along with alterations in the tear film composition [22]. Notably, the protein content of the tear film is altered, and elevated cytokine levels have been observed, indicating underlying inflammation [23,24]. AAK is a progressive condition characterized by epithelial thinning or loss, chronic inflammation, and corneal vascularization, ultimately leading to corneal opacification. AAK has traditionally been attributed to LSCD [24]; however, increasing evidence suggests that it may primarily result from *PAX6* pathogenic variants, which affect multiple developmental pathways throughout the eye [25]. Additional ocular surface changes observed in aniridia include reduced corneal sensitivity, loss of subbasal nerve density, and increased presence of dendritic cells, reflecting neuroinflammatory dysfunction [26,27]. Corneal thickness is also abnormally increased in these patients [26]. While the anterior corneal layers are predominantly affected in AAK, signs of chronic epithelial defects and impaired wound healing are common [28]. Animal models further support these findings, showing an abnormally thin corneal epithelium [29] and a thickened corneal stroma [30]. Moreover, structural abnormalities of the limbal region, including inflammation, vascularization, and nerve fiber loss, have been linked to AAK progression. These are often accompanied by the presence of conjunctival tissue invading the corneal epithelium, indicating advanced LSCD [31]. Although neovascularization is not unique to aniridia, it frequently arises as a consequence of chronic inflammation, persistent epithelial defects, and limbal stem cell failure. More than half of individuals with congenital aniridia develop glaucoma, largely due to abnormal development of the trabecular meshwork and Schlemm’s canal [32].

The aim of our study was to measure OST at the corneal center and at points located 2 mm nasally, superiorly, temporally, and inferiorly from the corneal center in patients with congenital aniridia, and to compare these values with those of healthy individuals. Additionally, we sought to identify factors that may influence OST in individuals with congenital aniridia. Our findings may contribute to a deeper understanding of ocular surface alterations associated with this condition.

## 2. Patients and Methods

All examinations were conducted in accordance with the principles of the Declaration of Helsinki. The study was approved by the Regional and Institutional Committee of Science and Research Ethics of Semmelweis University (approval no. 80/2020, Supporting Information Files S1 and S2), and written informed consent was obtained from all participants, or from legal guardians in the case of underage patients.

Aniridia patients were identified through the Medsolution System (T-Systems Magyarország Zrt., Budapest, Hungary) at the Department of Ophthalmology, Semmelweis University. In the Medsolution System, between October 2005 and October 2022, a total of 88 patients with congenital aniridia were identified. Patient recruitment and examinations were conducted between 1 June 2022 and 30 September 2022. Exclusion criteria were (1) patients with high-amplitude nystagmus that prevented accurate image capture and (2) patients with ptosis that obscured or closely approached the defined ocular surface measurement points (2 mm from the center). The control group comprised randomly selected healthy individuals.

First, body temperature was measured using an infrared forehead thermometer. Following this, each participant completed the Ocular Surface Disease Index (OSDI) questionnaire. For each subject, the total OSDI score, vision-related subscore (derived from questions related to vision and task performance), and discomfort-related subscore (derived from questions addressing ocular surface discomfort) were calculated as described by Mathews et al. [33]. OSDI scores were categorized into severity ranges: normal (0–12), mild (13–22), moderate (23–32), and severe (33–100) ocular surface disease.

While participants completed the questionnaire, their OST adapted to the ambient conditions of the standardized examination room. OST was then measured using the TG-1000 Ocular Surface Thermographer (Tomey Corp., Nagoya, Japan). During the measurements, room conditions were standardized with an average temperature of 29.13 ± 2.01 °C and humidity of 42.2 ± 5.24%. To minimize environmental influence on OST, doors and windows remained closed, as air movement has been reported to significantly affect OST [3].

Measurements followed the protocol described by Mori et al. [34]. Participants blinked normally, closed both eyes for 5 s, and then kept their eyes open for at least 10 s. During the test, participants’ heads were stabilized using a standard ophthalmic chin and forehead rest, and they were instructed to fixate straight ahead. For each eye, a series of 11 OST images was captured at 1 s intervals, beginning at eye opening and continuing for 10 s. The temporally and spatially resolved temperature data were imported into MATLAB, 2023b (The MathWorks Inc., Natick, MA, USA) for further analysis. The Tomey Ocular Surface Thermographer measures OST over 11 s period, acquiring one image per second. Minor eye movements do not significantly affect the measurements; however, any manipulation of the ocular surface—such as eyelid holding or restriction of eye movement—may influence OST values. Therefore, no mechanical fixation was applied during our measurements. OST values from the first recording after eye opening were used for analysis. Recordings showing eyeball displacement were excluded. When the corneal center was not fully centrally aligned in the captured image, it was manually corrected before further data processing.

From the first image after eye opening, mean OST values were extracted at the corneal center and at locations 2 mm nasally, temporally, superiorly, and inferiorly from the corneal center. The average temperature at each point was calculated from the five nearest data points. In cases where the patient was unable to maintain perfect fixation and the device could not automatically determine the corneal center, the center was manually identified, and the surrounding measurement points were determined relative to that position. In a previous study, the TG-1000 ocular thermograph demonstrated high reproducibility in corneal surface temperature measurements at both central and mid-peripheral corneal locations under interobserver and intraobserver conditions. Cronbach’s alpha values were ≥0.90 across all measured corneal regions, indicating excellent measurement consistency [35]. In the present study, all OST measurements were performed by a single experienced examiner (O.N.).

Next, all participants underwent a comprehensive standard ophthalmological examination. We determined presence/absence of meibomian gland dysfunction (MGD), corneal erosions/ulcer, Salzmann nodular degeneration, aniridia-associated glaucoma, use of antiglaucomatous eye drops. In addition, patients with congenital aniridia were classified based on the degree of LSCD, the stage of AAK, and the severity of iris hypoplasia.

LSCD was graded according to previously published criteria as follows: no limbal changes; presence of an avascular pannus with less than 3 mm width; vascularized pannus with less than 3 mm width; and vascularized pannus exceeding 3 mm in width [31,36].

AAK was staged from 0 to 5: Stage 0 indicated no limbal changes; Grade 1 was defined by conjunctival tissue crossing the limbal border but extending no more than 1 mm onto the cornea; Grade 2 described a pannus extending across the peripheral cornea, typically involving the full 360 degrees; Grade 3 was characterized by pannus invasion of the central cornea, often covering it entirely with vessels; Grade 4 referred to a completely vascularized cornea; and Grade 5 represented end-stage disease, marked by a thick, opaque, and fully vascularized corneal surface [31,36].

Iris hypoplasia/malformation in patients with congenital aniridia was classified into four categories based on slit lamp examination without gonioscopy: atypical coloboma (grade 1), presence of more than 6 clock hours of iris remnants (grade 2), fewer than 6 clock hours of iris remnants (grade 3), and complete aniridia with no visible iris tissue (grade 4) [31,36]. AAK, LSCD, and iris malformation grading were performed by a single examiner (N.S.).

All patients underwent tomographic imaging using a rotating Scheimpflug camera (Pentacam HR, Oculus Optikgeräte GmbH, Wetzlar, Germany). From these examinations, the following corneal parameters were extracted: index of surface variance (ISV), index of surface asymmetry (IVA), index of height asymmetry (IHA), index of height decentration (IHD), central corneal thickness (CCT), pachymetry at the center of the pupil (PCP), pachymetry at the corneal apex (PCA), as well as pachymetry 2 mm superior, inferior, temporal, and nasal to the corneal center.

Statistical analysis was performed using SPSS software (version 19.0; IBM, New York, NY, USA). Age differences between groups were assessed using the Mann–Whitney U test. Sex distribution between the aniridia and control groups was compared using the chi-square test. Data from both eyes of each participant were included in the analysis. First, we performed an explorative univariate analysis. The Mann–Whitney U test was used to compare OSDI total and subscores (vision- and discomfort-related), OST (central and at all four paracentral positions), ISV, IVA, IHA, IHD, CCT, PCP, PCA and all above-described paracentral pachymetric parameters between the aniridia and control groups. Within the congenital aniridia group, using the Kruskal–Wallis test, we determined the effect of LSCD Grade, AAK Grade, iris malformation Grade and presence of Meibomian gland dysfunction on OST (central and at all four peripheral positions). Thereafter, using Pearson correlation analysis, we evaluated the effect of age on OST, as well as the relationship between corneal thickness and OST at the corresponding measurement locations. Using the Mann–Whitney U test, within the congenital aniridia group, we compared OST (central and at all four paracentral positions) between different AAK Grades, between patients without and with corneal erosion/ulcer, without and with Salzmann nodular degeneration, without and with aniridia associated glaucoma, without and with the use of anti-glaucoma eye drops, without and with the use of beta-blockers, carbonic anhydrase inhibitors, alpha-adrenergic agonists and prostaglandin analogues. We determined a *p*-value below 0.05 statistically significant. In case of multiple comparisons of OST across multiple locations and clinical subgroups, adjustment for multiple comparisons (Bonferroni correction) was applied.

Thereafter, we used a linear mixed-effects model (LMM) with subject identification number included as a random effect (avoiding treating both eyes from the same participant as independent observations). In this mixed-effects model, the covariate included age, the fixed effects included LSCD grade, AAK grade, iris malformation grade, presence of MGD, epithelial defects, Salzmann nodular degeneration, presence of glaucoma, prior use of antiglaucomatous eye drops and previous ocular surgery. Categorical variables were included as fixed effects using dummy coding, with the lowest category used as the reference. This allowed us to assess the impact of all fixed effects on OST at the different measurement locations. Statistical significance was defined as *p* < 0.05.

## 3. Results

The study included 45 eyes from 26 patients with congenital aniridia (mean age: 26.29 ± 17.78 years; range: 7–59; 55.6% female) (Supplementary Table S1). The control group comprised 47 eyes from 25 age-matched healthy individuals (mean age: 24.81 ± 4.73 years; range: 15–32; 68.1%; female; *p* = 0.163). Sex distribution did not differ significantly between the aniridia and control groups (χ^2^ = 1.05, *p* = 0.31).

Among congenital aniridia subjects, the analyzed 45 eyes of 26 persons belonged to 17 families. There were 11 (24.4%) eyes of 6 (23%) subjects with *PAX6* microdeletion, 9 (20%) eyes of 6 (23%) subjects with *PAX6* frameshift mutation, 9 (20%) eyes of 5 (19.2%) subjects with *PAX6* splicing mutation, 7 (15.5%) eyes of 4 (15.3%) subjects with *PAX6* nonsense mutation and 1 (2.2%) eye of 1 (3.8%) subject with *PAX6* missense mutation. In addition, in one family, regarding 8 (17.7%) eyes of 4 (15.3%) subjects, a microdeletion affecting a regulatory region has been verified.

The mean body temperature was 36.78 ± 0.46 °C in the congenital aniridia group and 36.83 ± 0.36 °C in the age-matched control group, with no significant difference between those (*p* = 0.606). Interestingly, OST, both centrally and at all four paracentral positions, showed a significant correlation with subject age (*p* ≤ 0.031). This effect was further evaluated using a linear mixed-effects model (see below).

Regarding LSCD grading among eyes with congenital aniridia, 2 (4.4%) eyes were classified as Grade 0, 1 (2.2%) eye as Grade 1, 21 (46.7%) eyes as Grade 2, 3 (6.7%) eyes as Grade 3, and 18 (40.0%) eyes as Grade 4.

For AAK staging, 3 (6.7%) eyes were classified as Grade 0, 15 (33.3%) eyes as Grade 1, 14 (31.1%) eyes as Grade 2, 5 (11.1%) eyes as Grade 3, and 8 (17.8%) eyes as Grade 4. No eyes were categorized as Grade 5.

Regarding iris malformation, 3 (6.7%) eyes were classified as Grade 0, 8 (17.8%) eyes as Grade 1, 13 (28.9%) eyes as Grade 2, 4 (8.9%) eyes as Grade 3, and 15 (33.3%) eyes as Grade 4. In 2 (4.1%) eyes, the iris malformation grade could not be determined due to corneal opacification.

Additional ocular surface findings in the aniridia group included meibomian gland dysfunction in 23 (51.1%) eyes, epithelial defects in 1 (2.2%) eye, and Salzmann’s nodular degeneration in 10 (22.2%) eyes.

In the congenital aniridia group, 27 (60.0%) eyes were diagnosed with glaucoma. Of these, 25 (92.59%) eyes were treated with β-blockers, 23 (85.18%) with carbonic anhydrase inhibitors, 9 (33.33%) with prostaglandin analogs, and 2 (7.4%) with α-adrenergic agonists. None of the control participants had glaucoma or were using ocular medications.

Some patients with congenital aniridia had undergone ocular surgery during their lifetime -the most recent surgical intervention had occurred 12 years prior to study commencement. Notably, none of the included eyes had a history of corneal surgery (0%). Phacoemulsification with in-the-bag intraocular lens implantation had been performed in 14 eyes of 7 patients (31.1%). Among these, one patient had additionally received simultaneous artificial iris implantation in both eyes (4.4%). Furthermore, both eyes of one patient had undergone lens removal without intraocular lens implantation (4.4%). No eye had undergone previous antiglaucomatous surgery (0%).

According to the OSDI classification, 33 (73.33%) eyes with aniridia were categorized as normal, 8 (17.78%) eyes as mild, and 2 (4.4%) eyes as moderate and 2 (4.4%) eyes as severe dry eye disease. In the control group, 41 (87.23%) eyes were classified as normal, 4 (8.89%) eyes as mild, and 2 (4.44%) eyes as moderate dry eye disease. There were no significant differences in OSDI total scores between patients with congenital aniridia and controls (9.19 ± 14.25 [range: 0–63.63] vs. 6.36 ± 6.83 [range: 0–22.92], *p* = 0.756), nor in the vision-related subscore (4.05 ± 8.34 [0–37.5] vs. 2.19 ± 4.14 [0–16.67], *p* = 0.498) or the discomfort-related subscore (5.49 ± 7.23 [0–27.05] vs. 4.16 ± 4.18 [0–14.5], *p* = 0.904). It is important to note that the OSDI questionnaire reflects the patient’s overall perception of visual function and ocular discomfort, without distinguishing between the better and worse eye. Therefore, interpretation of OSDI total and subscore data must account for potential bias introduced by the inclusion of both eyes from the same patient.

**Figure 1:**
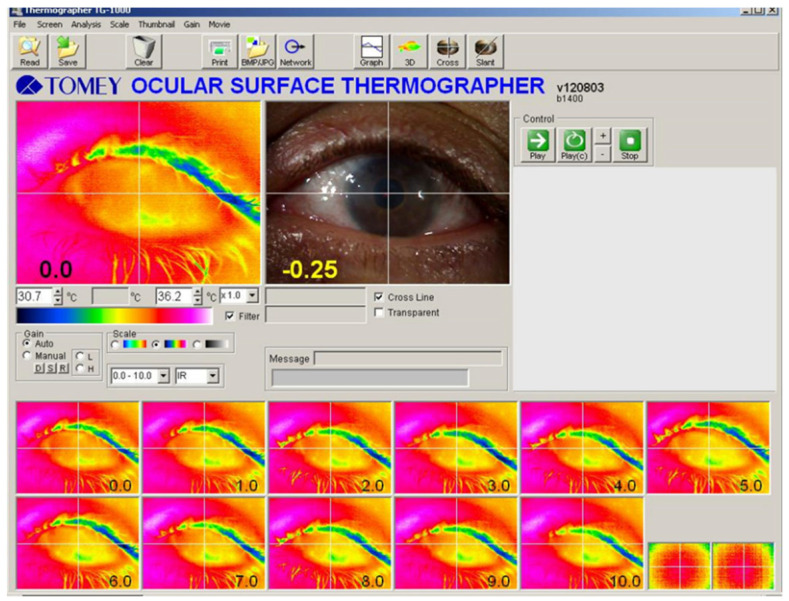
Ocular surface thermography image of a patient with congenital aniridia (patient following artificial iris implantation) during 10 s of sustained eye opening. The first image captured after eye opening was used to assess ocular surface temperature.

Ocular surface thermography images of a patient with congenital aniridia are shown in Figure 1. Table 1 summarizes the OST measurements in patients with congenital aniridia and in controls. The average central OST was 34.88 ± 0.59 °C in eyes with congenital aniridia and 34.73 ± 0.56 °C in controls, showing no significant difference between groups (*p* = 0.163). A significant difference was detected only in the superior OST (2 mm above the corneal center), which was higher in the aniridia group (34.98 ± 0.55 °C) compared to controls (34.75 ± 0.47 °C; *p* = 0.012). No significant differences were observed in the other regions—nasally, temporally, or inferiorly (all *p* ≥ 0.112).

Tomographic measurements of pachymetry, including CCT, PCP, PCA and at 2 mm superior, inferior, nasal, and temporal positions, are summarized in Table 2. All pachymetry measurements—including CCT, PCP, PCA, and those taken 2 mm from the corneal center in the superior, inferior, nasal, and temporal directions—were significantly higher in the aniridia group compared to controls (*p* < 0.001 for all). Nevertheless, no significant correlations were found between central OST and CCT, superior OST and superior pachymetry, inferior OST and inferior pachymetry, nasal OST and nasal pachymetry, or temporal OST and temporal pachymetry (*p* ≥ 0.105).

Additional corneal parameters—K1, K2, astigmatism, as well as tomographic indices including ISV, IVA, IHD, IHA—are presented in Table 3**.** K1 was significantly lower in the aniridia group (*p* < 0.001), while K2 showed no significant difference between groups (*p* = 0.383). Tomographic indices—ISV, IVA, IHD, and IHA—were all significantly elevated in the aniridia group relative to controls (*p* < 0.001), reflecting increased corneal irregularity.

**Figure 2:**
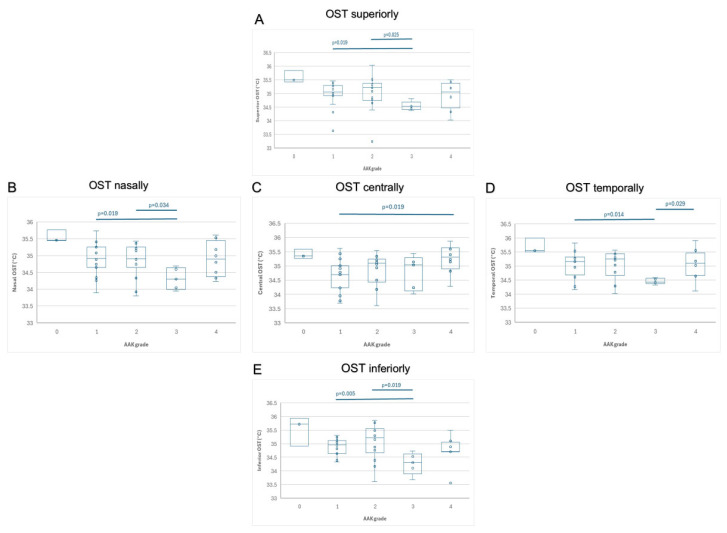
Within the group of patients with congenital aniridia, ocular surface temperature (OST) at the corneal center (**A**) and 2 mm from the center nasally (**B**), temporally (**C**), inferiorly (**D**), and superiorly (**E**) are displayed, stratified by aniridia-associated keratopathy (AAK) grades 0–4, according to Lagali et al. [27,32]. For AAK grade 0, the sample size was insufficient for statistical analysis. OST values of eyes with AAK grades 1–4 were compared at the central and all four peripheral positions using the Mann–Whitney U test. Central OST was significantly higher in AAK Grade 4, than in Grade 1 (*p* = 0.019). Nasal OST was significantly lower in AAK Grade 3, than in Grades 1 and 2 (*p* = 0.019; *p* = 0.034). Temporal OST was significantly lower in Grade 3, than in Grades 1 and 4 (*p* = 0.014; *p* = 0.029). Inferior OST was significantly lower in Grade 3, than in Grades 1 and 2 (*p* = 0.005; *p* = 0.019) and superior OST was significantly lower in Grade 3, than in Grades 1 and 2 (*p* = 0.019; *p* = 0.025).

Within the congenital aniridia group, using Kruskal–Wallis test, AAK Grade had a significant, negative effect on OST 2 mm below the corneal center (*p* = 0.03). Nevertheless, AAK Grade did not have an effect on OST at other corneal locations (*p* ≥ 0.114). Figure 2 shows the ocular surface temperature in different locations according to the aniridia-associated keratopathy grade. Central OST was significantly higher in AAK Grade 4 compared to Grade 1 (*p* = 0.019). At all peripheral positions, Grade 3 showed significantly lower OST values than Grades 1 and/or 2 (nasal: *p* = 0.019, *p* = 0.034; temporal: *p* = 0.014, *p* = 0.029; inferior: *p* = 0.005, *p* = 0.019; superior: *p* = 0.019, *p* = 0.025). Temporal OST was also lower in Grade 3 than in Grade 4 (*p* = 0.029).

In addition, iris malformation Grade had a significant, negative effect on OST at the corneal center (*p* = 0.020), superiorly (*p* = 0.029), nasally (*p* = 0.022) and temporally (*p* = 0.023). Nevertheless, when comparing central, superior, inferior, nasal, and temporal OST across the iris malformation grade subgroups, no significant differences were observed in any corneal region (*p* ≥ 0.200). Furthermore, LSCD Grade did not have a significant effect on OST at any positions (*p* ≥ 0.054). Presence of MGD or corneal erosion/ulcer also did not affect OST at any locations (*p* ≥ 0.166).

Within the congenital aniridia group, comparing subjects without and with Salzmann nodular degeneration, OST was significantly lower in eyes with Salzmann nodular degeneration at all corneal locations (*p* ≤ 0.011). Table 4 shows the ocular surface temperature in different locations in aniridia patients with and without Salzmann nodular degeneration.

Comparing OST (central and at all four paracentral positions) between patients without and with corneal erosion/ulcer, without and with aniridia associated glaucoma, without and with the use of antiglaucomatous eye drops, without and with the use of beta-blockers, carbonic anhydrase inhibitors, alpha-adrenergic agonists, prostaglandin analogues and without and with previous ocular surgery, OST did not differ significantly at any corneal locations (*p* ≥ 0.058).

In the linear mixed-effects model accounting for inter-eye correlation, age showed a consistent negative association with OST across most corneal locations, reaching nominal significance temporally (β = −0.02, *p* = 0.04), while trends were observed centrally and nasally that did not reach statistical significance (*p* = 0.09 and *p* = 0.06, respectively). No significant association between age and OST was observed at the superior or inferior locations (*p* ≥ 0.18) (Supplementary Table S2).

LSCD grade, AAK grade, and iris malformation grade were not significantly associated with OST at any corneal location in the mixed-effects model. Similarly, the presence of MGD and glaucoma, as well as the use of antiglaucomatous eye drops, showed no significant effects on OST at any measurement site (*p* ≥ 0.17).

The presence of epithelial defects was associated with a nominally significant reduction in temporal OST (β = −0.83, *p* = 0.01), while associations at other locations did not reach statistical significance (*p* ≥ 0.41).

In contrast, Salzmann nodular degeneration demonstrated a robust negative association with OST at all corneal locations, with the strongest effects observed superiorly (β = −1.49, *p* < 0.001) and temporally (β = −0.93, *p* = 0.003). Central, nasal, and inferior OST values were also significantly reduced in the presence of Salzmann nodular degeneration (all *p* ≤ 0.02).

Finally, previous ocular surgery was associated with a significant increase in OST at the central (β = 0.89, *p* = 0.03), nasal (β = 0.69, *p* = 0.02), and temporal (β = 0.81, *p* = 0.01) corneal locations, whereas no significant effects were observed superiorly or inferiorly (*p* ≥ 0.25) (Supplementary Table S2).

## 4. Discussion

The most conspicuous finding of our study is that OST 2 mm above the corneal center is significantly higher in eyes with congenital aniridia than in age-matched controls. In addition, AAK Grade has a significant, negative effect on OST 2 mm below the corneal center and that iris malformation grade has a significant, negative effect on OST at the corneal center, superiorly, nasally and temporally. Furthermore, Salzmann knots also significantly reduce OST at all corneal locations.

The presence of ocular surface disease has been shown to influence OST. AAK is characterized by abnormalities of the limbus, a variable degree of inflammation, and neovascularization, often accompanied by the presence of conjunctival tissue within the corneal epithelium [25]. Previous studies have reported elevated levels of inflammatory cytokines in tears of aniridia patients [23], which may be attributed to the inflammatory nature of AAK, chronic epithelial injury, or inflammation of the limbus and corneal stroma [26]. Despite this, ocular surface temperature has not previously been studied in patients with LSCD or aniridia. In other inflammatory ocular conditions, such as allergic conjunctivitis [13], infectious keratitis [14], and corneal transplant rejection [15], elevated OST has been observed, with temperature increases correlating directly with the degree of inflammation. In our study, the average central OST did not differ significantly between the aniridia and control groups. However, OST measured 2 mm superior to the corneal center was significantly higher in patients with aniridia, than in controls. Literature data describe in healthy individuals that OST tends to increase toward the limbus, likely due to the presence of limbal blood vessels [8]. Under inflammatory conditions, new blood vessels and conjunctival tissue migrate toward the central cornea, raising the local temperature. In aniridia, angiographic studies have shown that corneal neovascularization often originates at the 6 and 12 o’clock positions, sometimes appearing before it is visible with slit-lamp biomicroscopy [37]. This may explain the significantly higher OST 2 mm above the corneal center in congenital aniridia.

Comparing OST in eyes with different AAK Grades, the observed downward trend of OST with increasing AAK grade (in all corneal regions, except centrally, see Figure 2) is consistent with findings by Csorba et al. (2024), who noted a shift toward increased Langerhans cell maturity and decreased Langerhans cell density with advancing AAK grade, from Grade 2 AAK [27]. She suggested a reduction in active inflammation from Grade 2, which may contribute to the decline in ocular surface temperature as the disease progresses. Compared to Grade 1 AAK, OST showed a downward trend in Grade 3 AAK. Nevertheless, in Grade 4 keratopathy, OST increased again (Figure 2), most likely in parallel with the vascularization of the ocular surface in congenital aniridia.

Lagali et al. and Csorba et al. reported that the proportion of eyes with detectable central subbasal nerves significantly decreases as the AAK grade increases [27,31]. Csorba et al. (2024) found that in aniridia patients, corneal nerve fiber density, nerve fiber length, and total branch density were reduced, while nerve fiber width was increased in the subbasal nerve plexus [27]. These findings suggest that corneal innervation may play a critical role in the regulation of OST. Similar structural alterations in corneal nerves, accompanied by inflammation, have been documented in other ocular surface diseases. For example, in both Sjögren’s and non-Sjögren dry eye patients, Tepelus et al. (2017) observed a reduction in the subbasal nerve plexus together with an increased density of inflammatory dendritic cells [38]. This pattern may parallel the reduction in OST that we observed in Grade 3 AAK in our cohort.

In congenital aniridia, pathogenic variants in the *PAX6* gene lead to panocular developmental abnormalities, including structural and vascular alterations. All three components of the uveal vasculature—the iris, ciliary body, and choroid [39]—may be affected to varying degrees. Hypoplasia or aplasia of the iris and ciliary body are hallmark features of the disease [39]. The retinal vasculature in congenital aniridia also differs from that of healthy individuals, showing fewer retinal vessels, asymmetric vascular patterns, and reduced arching of the superficial vessels [40]. Optical coherence tomography angiography (OCTA) studies have revealed additional microvascular abnormalities, including absence of the foveal avascular zone, disruption of both superficial and deep vascular plexuses, and large superficial vessels abnormally diving into deeper layers [41]. Furthermore, Chen et al. (2020) reported significantly reduced subfoveal and parafoveal choroidal thickness in aniridic patients compared with healthy controls [42]. Whether similar mechanisms underlie uveal thinning in both the anterior and posterior segments in congenital aniridia remains unclear, as this phenomenon has not yet been systematically investigated. Nevertheless, such mechanisms could account for the significant negative correlation observed between iris malformation grade and OST across all corneal locations. Thus, both OST and iris malformation grade may serve as valuable biomarkers in congenital aniridia.

The OSDI questionnaire is a validated tool for distinguishing between normal, mild-to-moderate, and severe dry eye disease. Fries et al. [43] recently reported that, in patients with congenital aniridia, overall OSDI scores increase with advancing AAK grade. In contrast, our study found no significant differences in total OSDI score, vision-related subscore, or discomfort-related subscore between congenital aniridia patients and controls, and thus we could not confirm this association. One possible explanation lies in the limitations of the OSDI when applied to populations with reduced visual acuity. The tool’s validity and reliability were originally established in patients with relatively good vision—typically better than 0.5 decimal visual acuity, equivalent to “walking-around vision” [44]. In our aniridia group, mean best-corrected visual acuity (BCVA) was only 0.18 ± 0.13 [0.01–0.5]. Mathews et al. (2013) found that many glaucoma patients have ocular surface disease, with glaucoma treatment further increasing the risk [33]. They also observed that OSDI scores in glaucoma patients may not accurately reflect dry eye symptoms, as responses are often more closely related to visual field loss. This raises concerns about the interpretability of OSDI scores in low-vision populations or in those with visual field defects, such as congenital aniridia. In our cohort, 60% of eyes were diagnosed with glaucoma. Nevertheless, the discomfort-related OSDI subscore also did not differ significantly between congenital aniridia and control groups, suggesting that non–vision-related factors were similarly comparable between the two groups.

In congenital aniridia, multiple factors likely contribute to ocular surface alterations. Patients often present with dry eye disease [45], meibomian gland dysfunction [23] and changes in tear film composition [22], all of which may influence OST and overall ocular surface health. However, our data suggest that these factors do not significantly affect OST in eyes with congenital aniridia. In Salzmann nodular degeneration, prominent nodules can cause tear film instability and irregularity [46]. In our cohort, the presence of Salzmann nodules was associated with a significant reduction in OST across all corneal locations. Their surgical removal may therefore be beneficial in advanced cases to help maintain ocular surface homeostasis. Nonetheless, the wound healing process after such procedures must be carefully monitored, as it could inadvertently accelerate the progression of AAK. In addition, the potential impact of antiproliferative agents used intraoperatively—such as mitomycin C—on disease progression warrants further investigation [47].

Interestingly, neither the diagnosis of glaucoma (60% of eyes with congenital aniridia) nor the use of different antiglaucomatous eye drops had a significant effect on OST in eyes with congenital aniridia, suggesting that other factors may play a more prominent role in OST regulation in these patients. Secondary glaucoma develops in approximately 50–75% of individuals with congenital aniridia, often manifesting before adulthood, due to abnormal differentiation of the trabecular meshwork and Schlemm’s canal [32]. The relationship between OST and glaucoma remains controversial. Some studies have reported increased OST in glaucomatous eyes [18], while others found lower central OST following eye opening and a significantly faster decrease in surface temperature in glaucoma patients compared to healthy controls [19]. Studies by Gugleta and Galassi showed that although OST does not correlate with intraocular pressure (IOP), it does correlate with Doppler parameters of the ophthalmic and posterior ciliary arteries, suggesting that OST may serve as an indirect marker of impaired optic nerve head perfusion [7,20]. In glaucoma, reduced retrobulbar blood flow velocities [48], as well as altered retinal [49] and choroidal perfusion [50], have been reported. The medical management of glaucoma in patients with congenital aniridia does not differ significantly from that of other glaucoma types. However, the use of combination therapy is often necessary to achieve adequate IOP control [51]. It is important to note that topically applied antiglaucoma medications not only reduce IOP but can also affect ocular blood flow, including that of the ciliary body and other structures involved in regulating OST [52,53]. For example, Fuchsjäger-Mayrl et al. reported that dorzolamide increases blood flow in the optic nerve head and choroid in patients with primary open-angle glaucoma or ocular hypertension, whereas timolol showed no significant effect on ocular blood circulation [53]. Similarly, Konieczka et al. (2018) found that brimonidine, but not latanoprost, significantly affected central corneal temperature, likely due to reduced ocular blood flow induced by the medication [8]. In congenital aniridia, neither glaucoma nor the use of antiglaucomatous eye drops appears to significantly affect OST, suggesting that other factors may be responsible. Most probably, changes in uveal blood-flow may play a more decisive role in OST than glaucoma itself.

Corneal thickness is pathologically increased in aniridia, as previously described by Lagali et al. (2020), particularly in association with AAK [26,31]. Consistent with these findings, our study demonstrated that central corneal thickness, pachymetry at the pupil, apex, and 2 mm superiorly, inferiorly, nasally, and temporally from the corneal center were significantly higher in the aniridia group compared to controls. The impact of corneal thickness on OST remains controversial. Pattmöller et al. (2015) found no correlation between CCT and OST in healthy individuals [11]. In contrast, Aliò and Padrón (1982) observed a progressive increase in OST from the corneal center toward the periphery [54]. Purslow and Wolffsohn (2007) reported a weak negative correlation between corneal thickness and OST using the Thermo Tracer 7210MX [10]. In our study, central OST showed no significant association with CCT, nor did superior, inferior, nasal, or temporal OST correlate significantly with pachymetry measurements at their respective locations, indicating that corneal thickness has no significant influence on OST in congenital aniridia.

In this study, linear mixed-effects modeling revealed that OST is influenced primarily by age-related factors and specific corneal surface pathologies rather than by the severity of congenital aniridia–associated structural abnormalities. Age showed a consistent negative association with OST across several corneal locations, most prominently at the temporal site, supporting the concept that ocular surface physiology undergoes gradual thermoregulatory changes with aging, potentially related to alterations in tear film dynamics, vascular supply, or metabolic activity of the ocular surface. Notably, LSCD grade, AAK grade, and iris malformation grade were not independently associated with OST after accounting for inter-eye correlation and relevant covariates. These findings suggest that, while these disease-specific features reflect structural and functional impairment in congenital aniridia, they may not directly translate into measurable changes in ocular surface temperature, or their effects may be overshadowed by more dominant local surface conditions. This highlights the complexity of OST as a biomarker, which likely reflects a combination of epithelial integrity, inflammation, and surface homeostasis rather than disease severity alone. In contrast, Salzmann nodular degeneration emerged as the strongest and most consistent predictor of reduced OST across all corneal locations, indicating a substantial impact on ocular surface physiology. The pronounced temperature reduction observed in eyes with Salzmann nodules may reflect chronic stromal remodeling, reduced epithelial metabolism, or altered local perfusion, consistent with the long-standing and degenerative nature of this condition [47]. Similarly, the presence of epithelial defects was associated with lower temporal OST, supporting the notion that disruption of epithelial integrity and barrier function is accompanied by measurable thermal changes. Interestingly, previous ocular surgery was associated with higher OST at central and paracentral locations, which may indicate persistent subclinical inflammation [22], altered corneal innervation [27], or long-term changes in tear film composition following surgical intervention [22]. This finding underscores the importance of considering surgical history when interpreting OST measurements in both clinical and research settings. Taken together, these results suggest that OST is more sensitive to age, epithelial integrity, degenerative surface changes, and prior surgical intervention than to congenital aniridia–specific structural grading parameters. This reinforces the potential value of OST as a complementary functional biomarker of ocular surface health while also emphasizing the need for cautious interpretation in the context of multiple coexisting ocular surface conditions.

This study has limitations. Owing to the rarity of congenital aniridia, only 45 affected eyes were included. Severe corneal opacity prevented complete grading in all eyes, particularly with respect to iris malformation. In addition, patient-reported OSDI scores reflect overall ocular discomfort and cannot be attributed to individual eyes. Although aniridic corneas were thicker, corneal thickness was not associated with OST, leaving uncertainty regarding how structural alterations translate into ocular surface thermal patterns. Furthermore, the frequent presence of nystagmus in patients with congenital aniridia may complicate accurate OST measurement in routine clinical practice and may have introduced bias in the present study. Because congenital aniridia and AAK are progressive conditions, longitudinal studies are needed to determine whether changes in OST can predict disease progression and serve as an early, noninvasive marker of worsening ocular surface pathology. Finally, given the central role of inflammation and abnormal vascularization in congenital aniridia, further studies are required to clarify how limbal vascular changes relate to OST and to elucidate the vascular mechanisms underlying the observed thermal patterns. Future studies with larger sample sizes are warranted to further evaluate the potential of OST as a biomarker in congenital aniridia.

## 5. Conclusions

Our findings demonstrate that OST distribution is altered in eyes with congenital aniridia, with region-specific differences observed compared to age-matched controls. While exploratory univariate analyses suggested associations between increasing AAK severity, iris malformation grade, and reduced OST at multiple corneal locations, these relationships were not independently confirmed in multivariable linear mixed-effects modeling. In contrast, age, Salzmann nodular degeneration, and prior ocular surgery emerged as key determinants of OST, highlighting their dominant influence on ocular surface thermal patterns. Collectively, these results indicate that OST reflects ocular surface integrity and secondary surface pathology rather than aniridia-specific structural severity alone, supporting its potential role as a noninvasive functional biomarker when interpreted in appropriate clinical contexts.

## Figures and Tables

**Table 1:** Ocular surface temperature (OST, °C) was measured at the central cornea and at superior, inferior, nasal, and temporal positions located 2 mm from the corneal center. Data are presented as mean  ±  standard deviation (range: minimum–maximum). *p*-values represent comparisons between eyes with congenital aniridia and control eyes, analyzed using the Mann–Whitney U test. Statistically significant results (*p* < 0.05) are indicated in bold. A significant difference was observed only in the superior OST-measured 2 mm above the corneal center-which was significantly higher in the aniridia group compared to controls (*p* = 0.012).

	Central OST	Superior OST	Inferior OST	Nasal OST	Temporal OST
**Congenital Aniridia**	34.88 ± 0.59 (33.47–35.78)	34.98 ± 0.55 (33.24–36.03)	34.88 ± 0.56 (33.56–35.94)	34.86 ± 0.52 (33.80–35.77)	35.02 ± 0.51 (34.01–35.99)
**Control**	34.73 ± 0.56 (33.61–35.87)	34.75 ± 0.47 (33.77–35.68)	34.76 ± 0.59 (33.39–35.77)	34.77 ± 0.51 (33.77–35.76)	34.85 ± 0.49 (33.89–35.77)
***p*** **-value**	0.163	**0.012**	0.278	0.388	0.112

**Table 2:** Corneal thickness measurements at various regions are presented in micrometers (µm). Data are expressed as mean  ±  standard deviation (range: minimum–maximum). *p*-values represent comparisons between eyes with congenital aniridia and control eyes, analyzed using the Mann–Whitney U test. Statistically significant values (*p* < 0.05) are shown in bold. Corneal thickness was significantly greater in eyes with congenital aniridia at all measured locations (*p* < 0.001 for all comparisons).

	Central Corneal Thickness (CCT)	Pachymetry at the Pupil (PCP)	Pachymetry at the Apex (PCA)	Pachymetry 2 mm Superiorly	Pachymetry 2 mm Inferiorly	Pachymetry 2 mm Nasally	Pachymetry 2 mm Temporally
**Congenital Aniridia**	620.19 ± 63.73 (501–743)	654.21 ± 119.81 (508–1189)	649.24 ± 132.01 (473–1305)	664.43 ± 130.56 (104–1081)	680.62 ± 126.81 (537–1263)	682.41 ± 83.22 (548–959)	646.43 ± 128.04 (212–1161)
**Controls**	544.73 ± 38.89 (465–638)	546.80 ± 39.28 (469–643)	547.33 ± 39.38 (469–641)	600.71 ± 43.75 (507–703)	575.13 ± 41.62 (495–679)	589.27 ± 41.19 (513–684)	568.49 ± 41.10 (487–665)
***p*** **-value**	**<0.001**	**<0.001**	**<0.001**	**<0.001**	**<0.001**	**<0.001**	**<0.001**

**Table 3:** Corneal tomographic parameters—including K1 and K2, ISV (index of surface variance), IVA (index of vertical asymmetry), IHA (index of height asymmetry), and IHD (index of height decentration)—are presented for eyes with congenital aniridia and control eyes. Data are shown as mean  ±  standard deviation (range: minimum–maximum). *p*-values represent comparisons between groups using the Mann–Whitney U test. Statistically significant values (*p* < 0.05) are shown in bold. K1 values were significantly lower in the aniridia group compared to controls (*p* < 0.001), while no significant difference was observed in K2 (*p* = 0.383). All topographic indices—ISV, IVA, IHA, and IHD—were markedly elevated in aniridia eyes relative to controls, with all differences reaching statistical significance (*p* < 0.001).

	K1 (D)	K2 (D)	ISV	IVA	IHD	IHA
**Aniridia**	41.10 ± 2.43 (36.60–47.60)	43.70 ± 2.56 (39.00–48.70)	55.42 ± 40.55 (12–202)	0.49 ± 0.45 (0.08–1.79)	0.06 ± 0.16 (0.002–1.01)	13.89 ± 15.50 (1.1–64.4)
**Controls**	43.06 ± 1.68 (39.80–46.30)	44.06 ± 1.65 (40.70–46.90)	16.09 ± 4.93 (7–26)	0.11 ± 0.05 (0.03–0.22)	0.01 ± 0.005 (0.002–0.023)	4.74 ± 4.3 (0.8–12.3)
***p*** **-value**	**0.0001**	0.383	**<0.001**	**<0.001**	**<0.001**	**<0.001**

**Table 4:** The results of ocular surface temperature measurement in patients diagnosed with aniridia, categorised according to the presence or absence of Salzmann nodular degeneration. Data are presented as mean  ±  standard deviation (range: minimum–maximum). *p*-values represent comparisons between eyes with congenital aniridia patients, with and without Salzmann nodular degeneration analyzed using the Mann–Whitney U test. Statistically significant results (*p* < 0.05) are indicated in bold. Significant difference was observed in all of the measured localizations (*p* < 0.01).

	Central OST	Superior OST	Inferior OST	Nasal OST	Temporal OST
**With Salzmann nodular degeneration (n = 10)**	34.38 ± 0.54 (33.61–35.40)	34.52 ± 0.64 (33.24–35.51)	34.43 ± 0.73 (33.56–35.78)	34.48 ± 0.53 (33.80–35.43)	34.58 ± 0.46 (34.01–35.45)
**Without Salzmann nodular degeneration (n = 31)**	35.03 ± 0.53 (33.69–35.88)	35.11 ± 0.46 (33.63–36.03)	35.03 ± 0.46 (34.17–35.94)	34.97 ± 0.47 (33.89–35.77)	35.14 ± 0.48 (34.15–35.99)
***p*** **-value**	**0.005**	**0.01**	**0.01**	**0.01**	**0.007**

## Data Availability

The original contributions presented in this study are included in the article. Further inquiries can be directed to the corresponding author(s).

## Supplementary Materials

The following supporting information can be downloaded at https://www.mdpi.com/article/10.3390/life16020238/s1, Table S1: Demographic characteristics of the examined subjects.; Table S2: LMM.; Files S1 and S2: Institutional Review Board Approval Documents.

## Abbreviations

The following abbreviations are used in this manuscript:

AAK	aniridia-associated keratopathy
BCVA	best corrected visual acuity
CCT	central corneal thickness
IHA	index of height asymmetry
IHD	index of height decentration
ISV	index of surface variance
IVA	index of surface asymmetry
K1	corneal power (keratometry) at the flat meridian
K2	corneal power (keratometry) at the steep meridian
LSCD	limbal stem cell deficiency
MGD	meibomian gland dysfunction
OSDI	ocular surface disease index
OST	ocular surface temperature
*PAX6*	paired box 6 gene
PCA	pachymetry at the corneal apex
PCP	pachymetry at the center of the pupil
UCVA	uncorrected visual acuity
