# Supplementary material for: Altered Ocular Surface Temperature in Congenital Aniridia with PAX6 Pathogenic Variants: Impact of Age, Salzmann Nodules and Ocular Surgery
Source: Life (Basel). 2026 Feb 2;16(2):238. doi: 10.3390/life16020238 (PMC12941631; doi:10.3390/life16020238)
Supplement: Supplementary file 1 [file life-16-00238-s001.zip › Supplementary Table S1.pdf]

Patient Number	Group	Age (Years)	Gender (F=Female, M= Male)	Examined eye (R=right, L= Left)
1	Aniridia	7	F	R, L
2	Aniridia	7	M	R, L
3	Aniridia	8	M	L
4	Aniridia	10	M	R, L
5	Aniridia	10	M	L
6	Aniridia	10	F	R, L
7	Aniridia	11	F	R, L
8	Aniridia	12	F	R, L
9	Aniridia	13	F	R, L
10	Aniridia	14	M	R, L
11	Aniridia	15	F	R, L
12	Aniridia	15	M	R, L
13	Aniridia	15	F	R
14	Aniridia	16	M	L
15	Aniridia	22	M	R
16	Aniridia	24	M	R
17	Aniridia	25	M	R, L
18	Aniridia	39	M	R, L
19	Aniridia	41	F	R, L
20	Aniridia	41	F	R, L
21	Aniridia	44	F	R, L
22	Aniridia	47	M	R, L
23	Aniridia	49	F	R, L
24	Aniridia	50	M	R
25	Aniridia	60	F	R, L
26	Aniridia	60	F	R, L
27	Control	15	F	R, L
28	Control	16	F	R, L
29	Control	19	F	R, L
30	Control	19	F	R, L
31	Control	20	F	R, L
32	Control	20	M	R, L
33	Control	20	F	R
34	Control	20	F	R, L
35	Control	25	M	R, L
36	Control	25	M	L
37	Control	25	F	R
38	Control	25	M	R, L
39	Control	26	F	R, L
40	Control	27	M	R, L
41	Control	27	F	R, L
42	Control	28	F	R, L
43	Control	28	F	R, L
44	Control	28	M	R, L
45	Control	28	M	R, L
46	Control	28	M	R, L
47	Control	29	F	R, L
48	Control	29	F	R, L
49	Control	29	F	R, L
50	Control	30	F	R, L
51	Control	32	F	R, L

Supplementary Table 1. Demographic characteristics of the examined subjects.
