# Supplementary material for: Altered Ocular Surface Temperature in Congenital Aniridia with PAX6 Pathogenic Variants: Impact of Age, Salzmann Nodules and Ocular Surgery
Source: Life (Basel). 2026 Feb 2;16(2):238. doi: 10.3390/life16020238 (PMC12941631; doi:10.3390/life16020238)
Supplement: Supplementary file 1 [file life-16-00238-s001.zip › Supplementary Table S2.pdf]

Supplementary Table 2. Linear mixed-effects model (LMM) analysis of factors associated with ocular surface temperature (OST) measured centrally and at four paracentral corneal locations. Age was a covariate, fixed effects included limbal stem cell deficiency (LSCD) grade, aniridia-associated keratopathy (AAK) grade, iris malformation grade, presence of meibomian gland dysfunction (MGD), epithelial defects, Salzmann nodular degeneration, glaucoma, use of antiglaucomatous eye drops, and previous ocular surgery. Subject identification number was included as a random effect to account for inter-eye correlation. Results are presented as regression coefficients (β), standard errors (SE), 95% confidence intervals (CI), t values, and corresponding p values. Negative β values indicate lower OST relative to the reference category. LSCD Grade 2, AAK Grade 0 and Iris Grade 1 were used as reference categories. LSCD grade 0 and Iris Grade 0 were excluded from the linear mixed-effects model due to the very small number of eyes in these categories. The effect of antiglaucomatous eye-drops use could not be estimated in the model due to lack of variability and/or collinearity with glaucoma status (0 values below).

Parameter	*OST*	*Estimate (β)*	*Standard error (SE)*	*95% CI*	*t*	*p values*
Age	*OST central*	-0.03	0.01	-0.06-0.01	-1.89	0.09
	OST nasal	-0.02	0.01	-0.04-0.001	-2.11	0.06
	OST temporal	-0.02	0.01	-0.05- -0.002	-2.40	***0.04***
	OST superior	-0.02	0.02	-0.06-0.01	-1.46	0.18
	OST inferior	-0.01	0.01	-0.04-0.03	-0.35	0.73
LSCD Grade 1	*OST central*	-0.21	0.66	-1.69-1.26	-0.32	0.76
	OST nasal	-0.09	0.49	-1.16-0.97	-0.2	0.85
	OST temporal	-0.20	0.48	-1.28-0.88	-0.42	0.69
	OST superior	-0.07	0.71	-1.67-1.53	-0.1	0.92
	OST inferior	-0.90	0.66	-2.39-0.59	-1.37	0.20
LSCD Grade 3	*OST central*	0.26	0.61	-1.09-1.6	0.42	0.68
	OST nasal	0.09	0.48	-0.94-1.13	0.20	0.85
	OST temporal	0.26	0.46	-0.73-1.26	0.58	0.57
	OST superior	0.07	0.65	-1.38-1.51	0.11	0.92
	OST inferior	0.25	0.61	-1.12-1.61	0.40	0.69

LSCD Grade 4	*OST central*	-0.56	0.62	-1.94-0.33	-0.92	0.38
	OST nasal	-0.67	0.46	-1.67-0.59	-1.47	0.17
	OST temporal	-0.64	0.45	-1.65-0.37	-1.41	0.19
	OST superior	-0.29	0.66	-1.78-1.20	-0.44	0.67
	OST inferior	-0.61	0.62	-2.00-0.79	-0.98	0.35
AAK Grade 1	*OST central*	-0.63	0.58	-1.97 -0.70	-1.10	0.31
	OST nasal	-0.54	0.39	-1.45-3.04	-1.35	0.22
	OST temporal	-0.53	0.42	-1.50-0.38	-1.26	0.24
	OST superior	-0.45	0.64	-1.90-1.00	-0.71	0.49
	OST inferior	-0.89	0.58	-2.25-0.46	-1.54	0.17
AAK Grade 2	*OST central*	-0.81	0.89	-2.84-1.21	-0.90	0.39
	OST nasal	-0.49	0.64	-1.92-0.94	-0.79	0.46
	OST temporal	-0.54	0.65	-2.01-0.94	-0.89	0.43
	OST superior	-0.23	0.97	-2.42-1.97	-0.23	0.82
	OST inferior	-0.75	0.89	-2.8-1.30	-0.84	0.43
AAK Grade 3	*OST central*	0.46	1.37	-2.59-3.51	0.34	0.74
	OST nasal	0.77	1.04	-1.5-3.04	0.74	0.48
	OST temporal	0.73	1.02	-1.52-2.98	0.72	0.49
	OST superior	1.18	1.47	-2.11-4.48	0.80	0.44
	OST inferior	0.21	1.38	-2.88-3.29	0.15	0.88
AAK Grade 4	*OST central*	0.02	1.41	-3.08-3.13	0.02	0.98
	OST nasal	0.069	1.08	-1.67-3.05	0.63	0.54
	OST temporal	0.53	1.05	-1.77-2.84	0.51	0.62
	OST superior	0.78	1.05	-2.6-4.11	0.52	0.62
	OST inferior	-0.15	1.41	-3.29-2.99	-0.11	0.92
Iris Grade 2	*OST central*	-0.10	0.36	-0.91-0.52	0.7	0.78
	OST nasal	-0.24	0.26	-0.81-0.32	-0.97	0.36

	OST temporal	-0.21	0.26	-0.8-0.37	-0.82	0.43
	OST superior	-0.09	0.39	-0.96-0.79	-0.23	0.82
	OST inferior	-0.29	0.36	-1.12-0.52	-0.84	0.42
Iris Grade 3	*OST central*	-0.31	0.56	-1.58-0.98	-0.54	0.60
	OST nasal	-0.32	0.39	-1.21-0.56	-0.84	0.42
	OST temporal	-0.38	0.41	-1.31-0.55	-0.93	0.38
	OST superior	-0.33	0.61	-1.72-1.06	-0.54	0.61
	OST inferior	-0.23	0.56	-1.53-1.07	-0.41	0.69
Iris Grade 4	*OST central*	0.38	0.44	-0.62-1.37	0.85	0.42
	OST nasal	0.02	0.32	-0.82-0.32	0.07	0.95
	OST temporal	0.15	0.32	-0.58-0.89	0.47	0.65
	OST superior	-0.05	0.48	-1.12-1.03	-0.10	0.92
	OST inferior	0.05	0.44	-0.95-1.06	0.11	0.91
MGD	*OST central*	0.16	0.39	-0.69-1.01	0.41	0.69
	OST nasal	0.16	0.32	-0.52-0.83	0.5	0.62
	OST temporal	0.12	0.29	-0.51-0.75	0.41	0.69
	OST superior	0.16	0.41	-0.75-1.07	0.39	0.70
	OST inferior	0.11	0.39	-0.75-0.97	0.27	0.79
Epithelial defects	OST central	-0.73	0.33	-1.45- -0.02	-2.20	0.44
	OST nasal	-0.63	0.45	-1.56-0.32	-1.40	0.18
	OST temporal	-0.83	0.29	-1.47- -0.19	-2.78	***0.01***
	OST superior	-0.27	0.32	-0.95-0.41	-0.84	0.41
	OST inferior	-0.70	0.36	-1.47-0.07	0.27	0.79
Salzmann nodular degeneration	OST central	-0.85	0.31	-1.51- -0.18	-2.69	***0.02***
	OST nasal	-1.05	0.38	-1.82- -0.27	-2.78	***0.01***
	OST temporal	-0.93	0.27	-1.51- -0.36	-3.41	***0.003***
	OST superior	-1.49	0.30	-2.14-0.85	-4.91	***<0.001***

	OST inferior	-1.03	0.33	-1.73- -0.32	-3.07	***0.007***
Glaucoma	OST central	-0.34	0.47	-1.39-0.71	-0.72	0.49
	OST nasal	-0.14	0.33	-0.89-0.60	-0.43	0.67
	OST temporal	-0.2	0.34	-0.97-0.56	-0.60	0.56
	OST superior	-0.26	0.50	-1.4-0.88	-0.51	0.62
	OST inferior	0.27	0.46	-0.79-1.34	0.58	0.58
Use of antiglaucomatous eye-drops	OST central	0	0	0	0	0
	OST nasal	0	0	0	0	0
	OST temporal	0	0	0	0	0
	OST superior	0	0	0	0	0
	OST inferior	0	0	0	0	0
Surgery	OST central	0.89	0.36	0.10-1.69	2.53	***0.03***
	OST nasal	0.69	0.26	0.12-1.26	2.65	***0.02***
	OST temporal	0.81	0.26	0.23-1.39	3.13	***0.01***
	OST superior	0.47	0.38	-0.39-1.32	1.22	0.25
	OST inferior	-0.08	0.35	-0.89-0.72	-0.24	0.81
