# Supplementary material for: Altered Ocular Surface Temperature in Congenital Aniridia with PAX6 Pathogenic Variants: Impact of Age, Salzmann Nodules and Ocular Surgery
Source: Life (Basel). 2026 Feb 2;16(2):238. doi: 10.3390/life16020238 (PMC12941631; doi:10.3390/life16020238)
Supplement: Supplementary file 1 [file life-16-00238-s001.zip › Supporting Information File S1.pdf]

Oktatás, kutatás, gyógyítás: 250 éve
az egészség szolgálatában

SEMMELWEIS EGYETEM
Regionális, Intézményi Tudományos és Kutatásetikai Bizottság
Elnök: Prof. Dr. Sótonyi Péter

SE RKEB szám: 80/2020.
Protokoll: -

Dr. Szentmáry Nóra
egyetemi docens
Szemészeti Klinika

Budapest

Tárgy: „Kongenitális aniridia genetikai és képalkotó diagnosztikai vizsgálata" című kutatás

Tisztelt Docens Asszony!

A Semmelweis Egyetem Regionális, Intézményi Tudományos és Kutatásetikai Bizottsága a 2020. április 27-én megtartott ülésén az alábbi döntést hozta:

A bizottság a kutatási tervet szakmai és etikai szempontból megfelelőnek, valamint az intézmény tárgyi és személyi feltételeit a kutatás végzésére alkalmasnak találta.

A bizottság fenti döntését az egészségügyről szóló 1997. évi CLIV. törvény és az emberen végzett orvostudományi kutatásokról szóló 23/2002. (V.9.) EüM rendelet alapján hozta.

Felhívjuk figyelmét az adatvédelemmel kapcsolatos jogszabályok szigorú betartására, az adatvédelmi felelős kijelölésére is. (1997. évi XLVII. törvény 21. §: az egészségügyi és a hozzájuk kapcsolódó személyes adatok kezeléséről és védelméről.
2011. évi CXII. törvény: az információs önrendelkezési jogról és az információszabadságról.)
Továbbá tájékoztatjuk, hogy a kutatás, vizsgálat vagy klinikai vizsgálat végzése során a GDPR-ben (*AZ EURÓPAI PARLAMENT ÉS A TANÁCS (EU) 2016/679 RENDELETE (2016. április 27.) a természetes személyeknek a személyes adatok kezelése tekintetében történő védelméről és az ilyen adatok szabad áramlásáról, valamint a 95/46/EK rendelet hatályon kívül helyezéséről)* **foglaltakat kell figyelembe venni, és annak rendelkezéseit betartani**.

Tájékoztatásul közöljük, a RKEB bármikor ellenőrizheti, hogy a kutatást, vizsgálatot a kutatási tervben és az engedélyben előírtaknak megfelelően végzik-e [23/2002. (V.9.) EüM rendelet 18. §].

A vizsgálat befejezését követően kérjük a Bizottság részére a jelentés megküldését.

Budapest, 2020. május 5.

Semmelweis Egyetem
Regionális, Intézményi
Tudományos és Kutatásetikai Bizottság
1091 Budapest, Üllői út 93.
[illegible] 215-5038 Fax: [illegible]
[illegible] 450 Budapest, [illegible]

Dr. Sótonyi Péter
egyetemi tanár

Cím: 1091 Budapest, Üllői út. 93.
Postacím: 1085 Budapest, Üllői út. 26.; 1428 Budapest, Pf. 2.
E-mail: titkarsag.kutatasetikai-bizottsag@semmelweis-univ.hu

Tel.: (06-1) 215-7300/53513
Fax: (06-1) 215-7300/53512
Web: http://semmelweis.hu/rkeb

A fenti SE RKEB számra szíveskedjenek minden esetben hivatkozni.
