# Supplementary material for: Altered Ocular Surface Temperature in Congenital Aniridia with PAX6 Pathogenic Variants: Impact of Age, Salzmann Nodules and Ocular Surgery
Source: Life (Basel). 2026 Feb 2;16(2):238. doi: 10.3390/life16020238 (PMC12941631; doi:10.3390/life16020238)
Supplement: Supplementary file 1 [file life-16-00238-s001.zip › Supporting Information File S2.pdf]

SEMMELWEIS UNIVERSITY
Regional, Institutional Scientific and Research Ethics Committee
Chair: Prof. Dr. Péter Sótonyi

SE RKEB number: 80/2020
Protocol: –

Dr. Nóra Szentmáry
Associate Professor
Department of Ophthalmology

Budapest

Subject: *“Genetic and imaging diagnostics of congenital aniridia”*

Dear Associate Professor,

At its meeting held on 27 April 2020, the Regional, Institutional Scientific and Research Ethics Committee of Semmelweis University made the following decision:

The Committee found the research plan to be appropriate from both professional and ethical perspectives, and considered that the institution’s material and personal resources were suitable for conducting the research.

This decision of the Committee was taken on the basis of Act CLIV of 1997 on Health, and Decree 23/2002 (V.9.) of the Ministry of Health on medical research conducted on humans.

We draw your attention to the fact that, in order to regulate data management in compliance with the law, a data protection officer must also be appointed (Act XLVII of 1997, Section 21, on the handling and protection of personal health data and related data; Act CXII of 2011 on the Right of Informational Self-Determination and Freedom of Information).
Furthermore, we inform you that, in the course of the research, study, or clinical trial, the provisions of the GDPR must be taken into account and observed (REGULATION (EU) 2016/679 OF THE EUROPEAN PARLIAMENT AND OF THE COUNCIL of 27 April 2016 on the protection of natural persons with regard to the processing of personal data and on the free movement of such data, repealing Directive 95/46/EC).

For your information, the RKEB may check at any time whether the research or study is being carried out in accordance with the research plan and the terms of the approval (Decree 23/2002 (V.9.) of the Ministry of Health, Section 18).

Upon completion of the study, we kindly request that a report be submitted to the Committee.

Budapest, 5 May 2020

(stamp and signature)

Prof. Dr. Péter Sótonyi
University Professor

Address: 1091 Budapest, Üllői út 93.
Postal address: 1085 Budapest, Üllői út 26.; 1428 Budapest, Pf. 72.
Tel.: (06-1) 215-7300/53513

Fax: (06-1) 303-1118
E-mail: titkarsag.kutatásetikai.bizottsag@semmelweis-univ.hu
Website: www.semmelweis.hu/rkeb

Please kindly refer to the above SE RKEB number in all correspondence.
